# Cisplatin-resistant triple-negative breast cancer subtypes: multiple mechanisms of resistance

**DOI:** 10.1186/s12885-019-6278-9

**Published:** 2019-11-04

**Authors:** David P. Hill, Akeena Harper, Joan Malcolm, Monica S. McAndrews, Susan M. Mockus, Sara E. Patterson, Timothy Reynolds, Erich J. Baker, Carol J. Bult, Elissa J. Chesler, Judith A. Blake

**Affiliations:** 1 The Jackson Laboratory, ME 04609 and Farmington, Bar Harbor, CT 06032 USA; 2 Baylor University, Waco, TX 76798 USA

**Keywords:** Triple-negative breast cancer, TNBC, Cisplatin, Cisplatin sensitivity, Cancer subtypes, Gene expression, Cancer genomics, Drug response, Functional genomics, Data mining

## Abstract

**Abstract:**

**Background:**

Understanding mechanisms underlying specific chemotherapeutic responses in subtypes of cancer may improve identification of treatment strategies most likely to benefit particular patients. For example, triple-negative breast cancer (TNBC) patients have variable response to the chemotherapeutic agent cisplatin. Understanding the basis of treatment response in cancer subtypes will lead to more informed decisions about selection of treatment strategies.

**Methods:**

In this study we used an integrative functional genomics approach to investigate the molecular mechanisms underlying known cisplatin-response differences among subtypes of TNBC. To identify changes in gene expression that could explain mechanisms of resistance, we examined 102 evolutionarily conserved cisplatin-associated genes, evaluating their differential expression in the cisplatin-sensitive, basal-like 1 (BL1) and basal-like 2 (BL2) subtypes, and the two cisplatin-resistant, luminal androgen receptor (LAR) and mesenchymal (M) subtypes of TNBC.

**Results:**

We found 20 genes that were differentially expressed in at least one subtype. Fifteen of the 20 genes are associated with cell death and are distributed among all TNBC subtypes. The less cisplatin-responsive LAR and M TNBC subtypes show different regulation of 13 genes compared to the more sensitive BL1 and BL2 subtypes. These 13 genes identify a variety of cisplatin-resistance mechanisms including increased transport and detoxification of cisplatin, and mis-regulation of the epithelial to mesenchymal transition.

**Conclusions:**

We identified gene signatures in resistant TNBC subtypes indicative of mechanisms of cisplatin. Our results indicate that response to cisplatin in TNBC has a complex foundation based on impact of treatment on distinct cellular pathways. We find that examination of expression data in the context of heterogeneous data such as drug-gene interactions leads to a better understanding of mechanisms at work in cancer therapy response.

## Background

A major goal of improved classification of cancer subtypes is to stratify patient populations and to more rapidly identify effective treatment strategies. Advances in molecular characterization of tumors not only improve classification, but also point directly to molecular mechanisms that lead to different therapeutic responses. By integrating heterogenous functional genomic data on tumor subtype characteristics, with known mechanisms and pathways and molecular response to drugs, it is possible to match drug response to tumor characteristics, thus refining treatment options.

### Subtypes of TNBC

Classification of cancer subtypes relies on many criteria including histological typing, mutation status, genomic structural variations and expression profiling [1–5]. Breast cancers are often classified by the presence or absence of three receptors: estrogen receptor (*ESR1*), progesterone receptor (*PGR*), and the HER2 epidermal growth factor receptor (*ERBB2*) [6, 7]. Tumors that lack expression of all three receptors are called triple-negative breast cancer (TNBC). As many available therapies in breast cancer target one of these receptors, TNBC status limits treatment options. TNBC is particularly aggressive with higher rates of recurrence, metastasis, and mortality than other breast cancers [8, 9].

Additionally, breast cancers are typically classified as luminal, basal/myoepithelial or ERBB2- subtypes based on relation to cell types found in the normal breast [10]. Although most TNBC cancers are characterized as basal-like, about 20% of TNBC tumors are classified as non-basal [11].

Two recent studies have classified TNBCs based on clustering genes that are up and down-regulated resulting in six and four molecularly defined subtypes, respectively [4, 5]. Lehmann et al. initially described and tested chemotherapy response in six TNBC subtypes: basal-like 1 (BL1), basal-like 2 (BL2), immunomodulatory (IM), mesenchymal (M), mesenchymal stem-like (MSL) and luminal androgen receptor (LAR) [4]. In another study, Burstein et al. also used gene-expression profiling to subclassify TNBC into four subtypes: mesenchymal (MES), luminal AR (LAR), basal-like immune suppressed (BLIS) and basal-like immune activated (BLIA) [5]. Burstein et al. compared their classifications with the Lehmann classifications and showed that there was some concordance with the LAR/LAR, MSL/MES and M/BLIS type tumors from both groups, but little discrimination of the BL1, BL2 and IM subtypes [5]. For our analysis, we used sets from four of the subtypes described by Lehmann et al: BL1, BL2, M, and LAR [12] (more details below).

### Treatment of TNBC

There are no targeted treatments for TNBC [13]. Standard treatment for TNBC patients includes chemotherapy and surgery and patients often become refractory to the treatment [14, 15]. Patients that achieve a complete response during neoadjuvant therapy generally have better outcomes [16]. Recent strategies for the treatment of TNBC define different treatments depending on *BRCA* gene status and *CD274* (PD-L1) expression status [17]. Treatments addressed include chemotherapy, immunotherapy, and PARP inhibitor therapy. First-line chemotherapeutic agents include taxane and anthracycline, which can be used singly or in combination, but these agents can be augmented with other treatments in cases of progression or contraindications [17].

### TNBC and Cisplatin

Although not currently considered standard of care for TNBC, there is renewed interest in cisplatin use to treat TNBC [18]. Cisplatin has been in use for over 40 years to treat multiple types of cancer. Substatial data correlating cisplatin sensitivity with respect to TNBC subtypes and curated data associating cisplatin with interacting genes provides a robust data collection for integrated analysis. This provides a unique opportunity to study the genetic mechanisms that underlie TNBC subtypes and their relation to cisplatin.

Currently, 22 clinical trials are exploring the use of cisplatin to treat TNBC either as a single agent or in combination with other therapies [19] (Search criteria were: not yet recruiting, recruiting, enrolling by invitation, and active, not recruiting accessed 01/22/2019). In particular, use of cisplatin therapy has been suggested for TNBC harboring a *BRCA* mutation [17]. Cisplatin is a DNA-intercalating agent that cross-links DNA resulting in interference with RNA transcription and DNA replication activities. If the DNA lesions are not repaired, DNA-damage induced cell-cycle arrest and apoptosis are triggered [20, 21]. Cells can become resistant to cisplatin by several mechanisms including change in the accumulation of the drug in cells either by inhibited uptake or enhanced efflux, detoxification of the drug by redox mechanisms, repair of the DNA by excision repair mechanisms, or negative regulation of apoptotic mechanisms [22–25].

*Relevance*

New insights into the biological processes associated with cisplatin in different molecular subtypes of TNBC may lead to [1] a better understanding of the mechanisms underlying treatment response differences, [2] strategies for identifying those patients that are more likely to respond robustly to chemotherapy, and [3] the identification of new treatment strategies.

### Approach

**Fig. 1:**
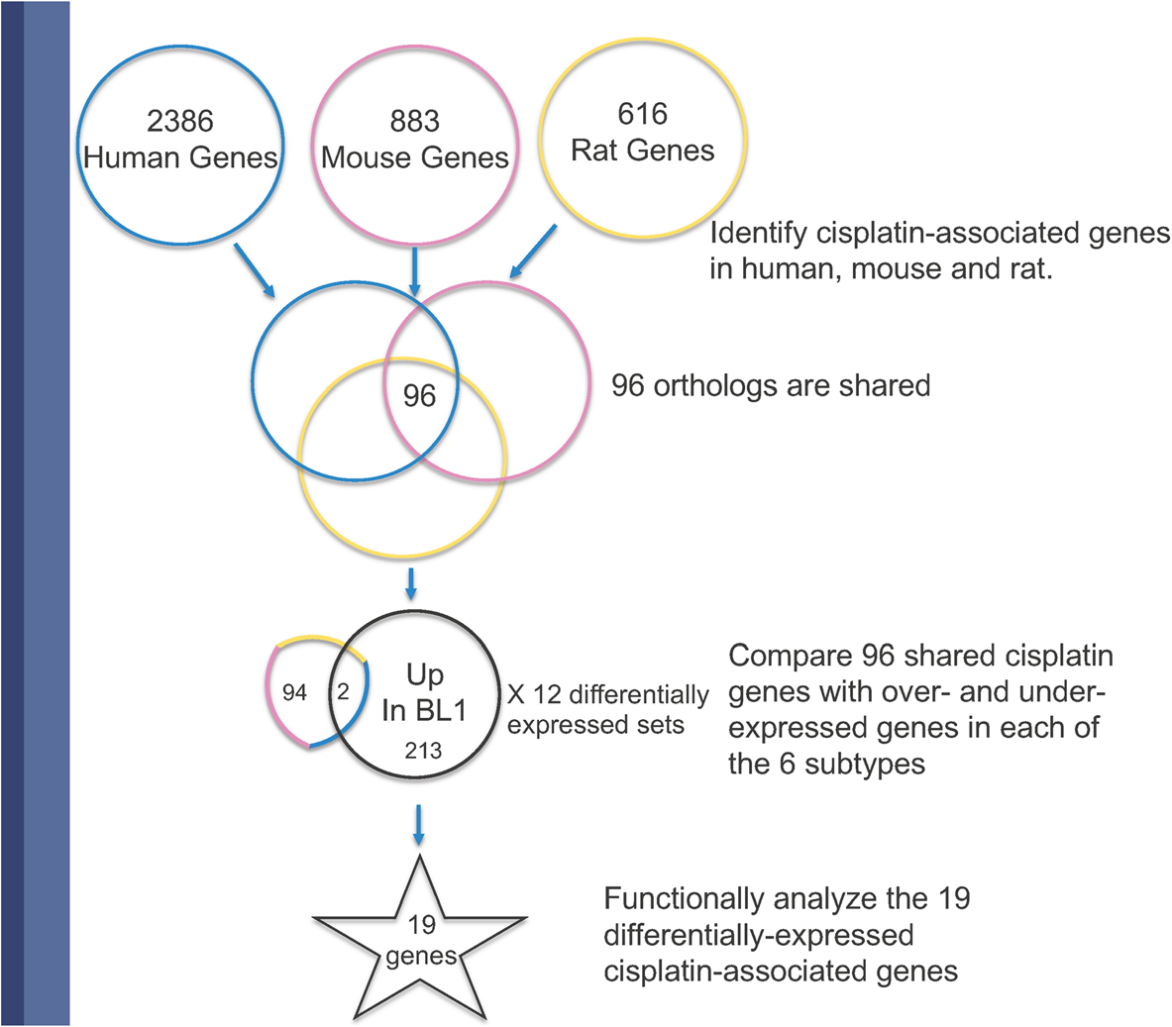
Title: Workflow to Identify Cisplatin-Related Processes in TNBC Subtypes. Legend: Summary of the strategy we used to identify cisplatin-related processes that are up and down-regulated in TNBC subtypes using the gene sets GS125959, GS257116 and GS263765. 1. Create a set of evolutionarily conserved genes that are associated with cisplatin. 2. Identify the conserved set of cisplatin-responsive genes that are differentially regulated in the TNBC subtypes. 3. Determine the GO biological processes and individual cisplatin-related processes that are enriched in the overlap set.

Our approach is to integrate and analyze curated information from pathways and mechanisms obtained in multiple species with empirical data collected in tumor profiling and mechanistic experiments. This allows us to focus, in a ‘sea’ of differentially expressed genes, on genes related to specific areas of interest--in our case genes related to the biology of cisplatin. In this study, we used the GeneWeaver (GW) gene set analysis platform [26] to identify specific biological processes that could explain the observation that of the TNBC subtypes, BL1 and BL2 are more sensitive to cisplatin than M and LAR [4]. We focus on these four subtypes because the MSL and IM subtypes were later shown to contain stromal cells and infiltrating lymphocytes respectively [12]. GW comprises a database of gene sets from multiple functional genomics data resources, curated publications and user submisisons. These data resources are provided with a suite of combinatorial and statistical tools for performing set operations on user selected gene lists. This provided a platform for the comparison of genomic profiles of multiple TNBC subtypes and gene products with a chemotherapeutic drug. To create the gene sets for our study we first identified evolutionarily conserved genes that were associated with cellular or physiological responses to cisplatin. We then identified which of the genes in the conserved cisplatin-associated set were found among genes shown previously to be differentially expressed in TNBC molecular subtypes. Finally, we analyzed the differentially expressed, cisplatin-associated genes with respect to biological processes and to pathways associated with sensitivity or resistance to cisplatin (Fig. 1).

## Methods

### Gene sets

To investigate these genes in the context of TNBC, we expanded the gene-set collection in GW by constructing genes sets for the differentially regulated genes described by Lehmann et al.*,* [4], thereby making gene sets for identified up- and down-regulated genes for each of the six molecular subtypes of TNBC. For our analysis, we used sets from four of the subtypes that were subsequently shown not to contain infiltrating cells: BL1, BL2, M, and LAR [12].

For all gene sets, we used Human Genome Nomenclature Committee (HGNC)-approved identifiers. Genes that we could not unambiguously assign to an HGNC identifier were not included. Details of the source and methods of curation are reported for each of the gene set descriptions as part of the GW record. For ontology-tagging, TNBC-gene sets were annotated with the Disease Ontology term ‘triple-receptor negative breast cancer’ (DOID:0060081), and the Human Phenotype Ontology term ‘Breast carcinoma’ (HP:0003002) ([27, 28], respectively). Gene sets with known response to cisplatin were tagged with the Chemicals of Biological Interest (ChEBI) term ‘cisplatin’ (CHEBI:27899) [29].

**Fig. 2:**
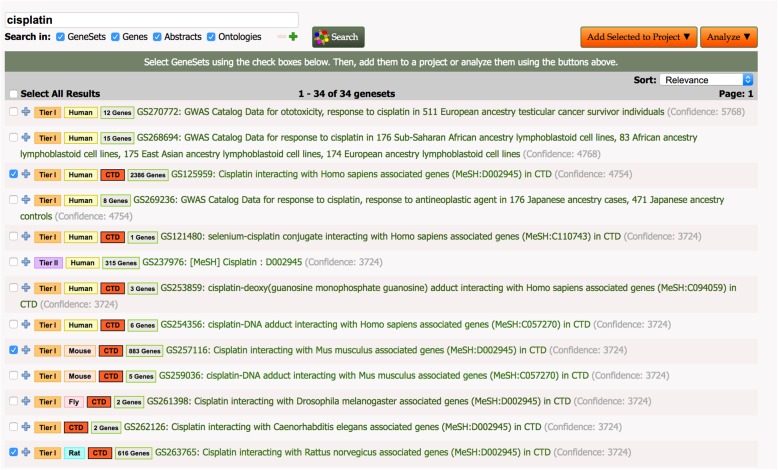
Title: GW Gene Sets Related to Cisplatin. Legend: A screen capture showing gene sets that match the string ‘cisplatin’ using the ‘GeneSet Search’ tool in GW. The search returned 34 sets of which the three selected sets were chosen to create our set of conserved genes. Title: Homologous Genes From Human, Mouse and Rat Related to Cisplatin. Legend: Results of the ‘HighSim’ graph tool in GW showing the number of genes in each of the gene sets derived from CTD at the top of the figure and the number of genes in each of the set intersections going to the bottom of the screen (analysis date 9/2/19). GeneWeaver gene-set identifiers for each of the intersections sets are shown below the boxes. The 96 genes resulting from the intersection of all three sets and the additional six from the MESH analysis comprise our set of conserved cisplatin-responsive genes. Abbreviations: H.s. = *Homo sapiens*, M.m. =*Mus musculus*, R.n. =*Rattus norvegicus*.

To create a set of human genes associated with cisplatin that are evolutionarily conserved, we identified gene sets associated with studies of cisplatin in GW’s database and applied combinatorial tools to selected sets as outlined below [30] (Fig. 2).

Using existing gene sets in GW we identified 34 cisplatin-associated gene sets that included sets obtained from GWAS studies (22 sets), MESH terms (2 sets) and the Comparative Toxicogenomics Database (CTD) (10 sets) respectively. CTD curates many aspects of gene-chemical interactions including regulatory, physical interaction, responses, and interactions that are reported as a result of interactions of cisplatin combined with other treatments [31]. The provenance of chemical-gene associations is fully traceable back to the original source. For eample the association of the gene *RAD51* with cisplatin can be traced back to three separate publications and three different species using the CTD resource (Query performed on Sept. 3, 2019).

We selected three large data sets from CTD for further analysis, one each from human, mouse and rat. The selected sets consisted of 2386 (GS125959), 883 (GS257116) and 616 (GS263765) genes from human, mouse and rat respectively. We chose these sets as ‘high-confidence’ sets because CTD data includes a large corpus of gene-chemical associations curated from published literature [32].

To identify genes associated with biological processes that are also evolutionarily conserved, and that therefore could be considered central to the action of cisplatin, we identified orthologous genes that share an association with cisplatin in CTD.

**Fig. 3:**
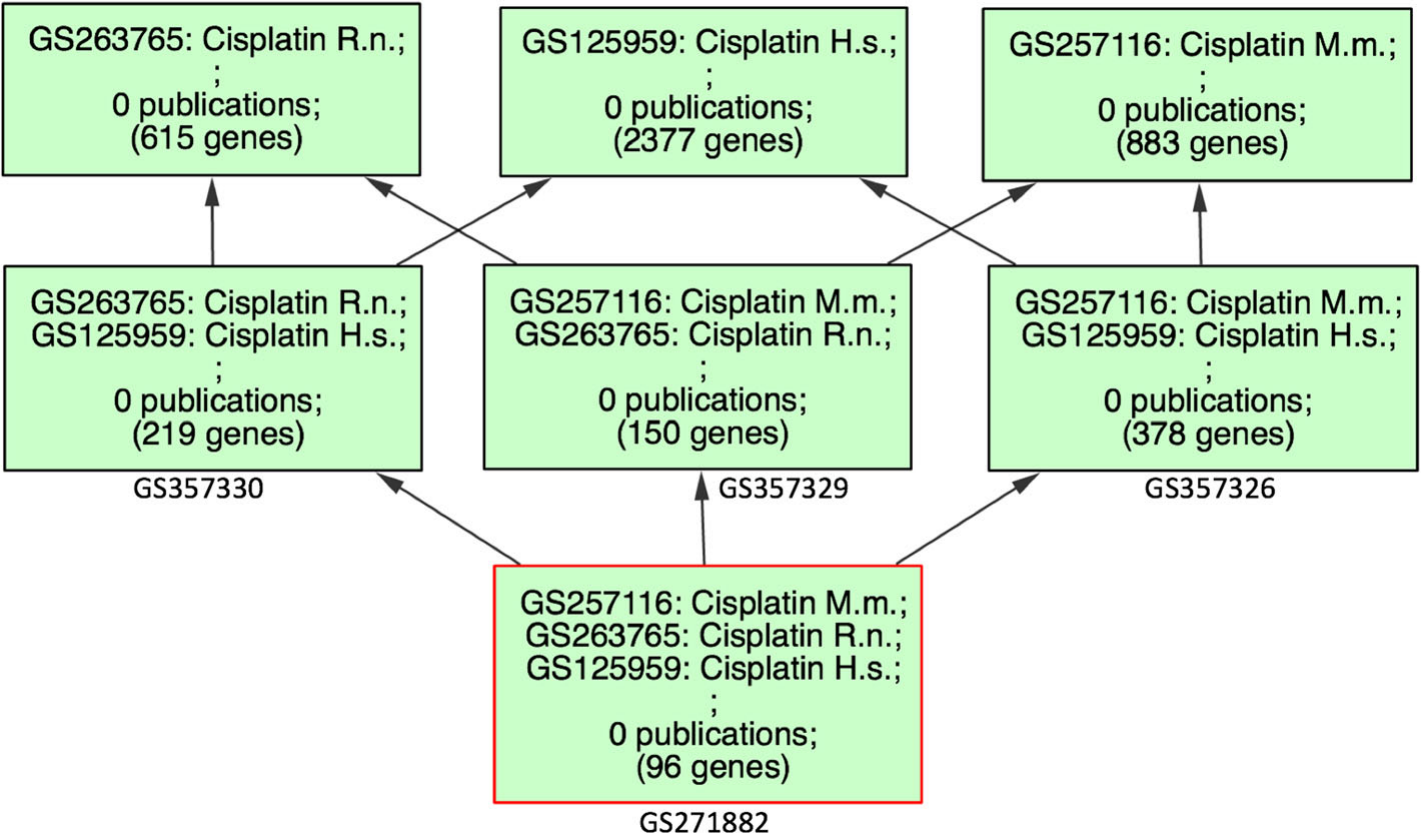
Title: Homologous Genes From Human, Mouse and Rat Related to Cisplatin Legend: Results of the ‘HighSim’ graph tool in GW showing the number of genes in each of the gene sets derived from CTD at the top of the figure and the number of genes in each of the set intersections going to the bottom of the screen (analysis date 9/2/19). GeneWeaver gene-set identifiers for each of the intersections sets are shown below the boxes. The 96 genes resulting from the intersection of all three sets and the additional six from the MESH analysis comprise our set of conserved cisplatin-responsive genes. Abbreviations: H.s. = *Homo sapiens*, M.m. =*Mus musculus*, R.n. =*Rattus norvegicus*.

To examine the orthologous gene overlap of these species-specific sets, we used the GW Hierarchical Similarity (HiSim) Graph tool [33]. This tool creates a graph in which leaves represent individual gene sets in the selection, and parent nodes represent sets of genes in the intersection of all child nodes (analysis date 9/2/19). Gene-overlap between mouse-human, rat-human and mouse-rat sets were 378, 219 and 151 genes respectively. We used the genes in the intersection of all three cisplatin-response sets to generate a new gene set of the 96 human cisplatin-associated genes whose homologs are conserved among the three species (GS271882) (Fig. 3). To supplement the data from the human CTD gene set, we performed the same analysis with an additional publically available gene set in GW, GS237976: [MeSH] Cisplatin:D002945. This analysis resulted in the identification of six more conserved genes: *GJA1, CCN1, H2AX, IL10, WRN, HSP90AA1*. Of these six genes only one, *GJA1*, was differentially expressed in the TNBC subtypes. We included these additional genes in our analysis. Gene Sets used for this study are listed in Table 1, for completeness we include sets for MSL and IM in this table but they were not used for further analysis.

**Table 1:** Gene Sets used for analysis in these studies. The first column is the Gene

GS ID	# of Genes	Gene Set Name
GS125959	2386	Cisplatin interacting with *Homo sapiens* associated genes (MeSH:D002945) in CTD
GS257116	883	GS257116: Cisplatin interacting with *Mus musculus* associated genes (MeSH:D002945) in CTD
GS263765	616	Cisplatin interacting with *Rattus norvegicus* associated genes (MeSH:D002945) in CTD
GS357326	378	Genes from CTD that interact with cisplatin and are conserved in human and mouse
GS357330	219	Genes from CTD that interact with cisplatin and are conserved in human and rat
GS357329	150	Genes from CTD that interact with cisplatin and are conserved in rat and mouse
GS271882	96	Genes from CTD that interact with cisplatin and are conserved in human, mouse and rat
GS237976	319	[MeSH] Cisplatin:D002945
GS271616	215	Genes upregulated in the BL1 subtype of triple negative breast cancer
GS271617	154	Genes upregulated in the BL2 subtype of triple negative breast cancer
GS271618	535	Genes upregulated in the IM subtype of triple negative breast cancer
GS271619	247	Genes upregulated in the M subtype of triple negative breast cancer
GS271621	805	Genes upregulated in LAR subtype of triple negative breast cancer
GS271724	829	Genes upregulated in the MSL subtype of triple negative breast cancer
GS271627	251	Genes downregulated in the BL1 subtype of triple negative breast cancer
GS271636	127	Genes downregulated in the BL2 subtype of triple negative breast cancer
GS271640	302	Genes downregulated in the IM subtype of triple negative breast cancer
GS271722	446	Genes downregulated in the M subtype of triple negative breast cancer
GS271729	382	Genes downregulated in the LAR subtype of triple negative breast cancer
GS271725	255	Genes downregulated in the MSL subtype of triple negative breast cancer

Weaver gene-set identifier and the second column is the number of genes in the set and the third column is the gene-set title.

### Gene set analysis

Gene sets were analyzed using the suite of tools from the GeneWeaver resource [26]. As described above, we used the ‘HiSim Graph’ tool to enumerate and visualize intersections among the gene sets from human, mouse and rat, and the ‘Boolean Algebra’ tool to create a set of conserved genes representing the intersection of the homologs of the three sets. We used the ‘Jaccard Similarity’ tool to statistically evaluate and identify genes in the gene-set overlap between the set associated with cisplatin treatment, and sets of over- and under-expressed genes in the TNBC subtypes. We used the default parameters for all analysis tools, details of which can be found at the GeneWeaver.org website [33].

### Gene function analysis

To identify processes enriched in gene sets and represent them in a graphical format we used the Visual Annotation Display (VLAD) tool for Gene Ontology enrichment analysis [34, 35]. First, to examine the 102 genes in the cisplatin-associated set we performed VLAD analysis to determine if those genes were enriched for processes known to represent cisplatin biology. We also tested the 20 cisplatin-associated genes that were differentially regulated in TNBC subtypes to see if their enrichment was different from the parental set, which would have indicated that those genes were enriched for a subset of processes that are involved in cisplatin biology. In all analyses, we used default parameters for VLAD enrichment analysis, and the set of UniProt-GOA human annotations as a background set [36]. The analysis was run on September 2, 2019. The UniProt-GOA gene annotation data used was dated from February 26, 2018. Since GO annotations represent processes that occur in normal cells and we are ultimately interested in the effects these genes have with respect to cisplatin treatment, we extended the functional characterization of the cisplatin-associated genes that are differentially regulated in resistant TNBC subtypes by manually searching for evidence describing how they might contribute to cisplatin reistance or sensitivity.

An additional functional analysis was performed with the 102 genes in the cisplatin-associated set using the KEGG Mapper Search Pathway tool to interrogate Pathways and Diseases [37]. Gene symbols were used with default parameters in the Organism-specific search mode (hsa). The analysis was performed on Sept 6, 2019.

We also ran an analysis using ‘String’, a network analysis tool that uses interaction data to functionally interrogate gene sets [38]. The analysis was performed on Sept 8, 2019. Genes were entered using gene symbols, analysis in human was selected and all default parameters were used. GO and KEGG catagories are reported from the ‘Functional Analysis’ results.

## Results

### Gene sets of differentially expressed genes in TNBC subtypes

To investigate sets of differentially regulated genes in TNBC subtypes, we created gene sets in GW for the six subtypes described by Lehmann et al [4]. We chose these subtypes because the Lehmann analysis includes a measure of relative sensitivity to cisplatin treatment. Using the information from the supplemental data in Lehmann et al, we associated their gene symbols with unique HGNC identifiers to create 12 gene sets: i.e., an up and down-expressed set for each of the six TNBC subtypes (Table 1) [39]. The gene sets ranged in size from 127 genes for which expression was down in the BL2 subtype, to 829 genes where expression is up in the MSL subtype. The 12 sets of up- and down-expressed genes represents 2161 unique human genes. Thirty-five genes were represented in 6 sets, and 101 genes were contained in 5 sets. One gene, *KRT17* (HGNC:6427), was listed in both the up- and down-expressed sets of MSL. For further analysis, we focused on the four TNBC subtypes that represent subtypes that only contain tumor-derived cells [12].

### Cisplatin-associated genes are enriched for processes that are consistent with the cytotoxic action and response to cisplatin

We hypothesized that by creating a gene set of evolutionarily-conserved cisplatin-interacting genes, we would select for genes that function in the fundamental actions of cisplatin. To test this, we used GO enrichment analysis to determine which biological processes were enriched in our 102 gene set. Our results confirm the validity of our strategy: we identified a set of genes that are involved in core cancer processes that are also known to be associated with action of cisplatin. Specifically, VLAD analysis showed that the 102 conserved cisplatin-associated genes were enriched for the GO biological processes: ‘aging’, ‘negative regulation of apoptotic process’, ‘apoptotic signaling pathway’, ‘response to ionizing radiation’, ‘cellular response to oxidative stress’, and ‘response to reactive oxygen species’ [Additional file 1: Table S1]. The 102 conserved genes were also enriched for the GO cellular component terms ‘chromosome, telomeric region’, ‘mitochondrion’, ‘cytosol’, ‘extracellular space’ and ‘membrane raft’ [Additional file 1: Table S1]. These results are consistent with the known mechanism of cisplatin action in which cisplatin causes oxidative stress, interacts with DNA and triggers a response that culminates in apoptosis [40].

We extended our GO results by interrogating the KEGG Pathway and KEGG Disease resources with the 102 conserved genes [41]. The KEGG Disease analysis showed that our genes were most represented in a variety of different cancer types with esophageal cancer associated with the most genes [5] [Additional file 2: Table S2]. DNA excision repair was associated with four genes and breast cancer was associated with two. The top scorerer for the KEGG Pathway mapping analysis was ‘cancer pathways’ (36 genes) [Additional file 3: Table S3]. KEGG pathway analysis was also consistent with, and confirmed the GO enrichment analysis: apoptosis (27 genes), cellular senescence (21 genes) and stress response pathways like the P53 pathway (20 genes). The KEGG analysis also identified several viral pathways as well as the platinum drug resistance class (22 genes) [Additional file 3: Table S3].

The set was interrogated using the String Network analysis tool [38]. Functional groupings from String were consistent with the VLAD and KEGG analysis results reported above [Additional file 4: Table S4].

### A subset of cisplatin-associated differentially-expressed genes provide a signature for the resistant subtypes

Of the 102 evolutionarily conserved cisplatin-assocated genes, 20 are differentially expressed in TNBC subtypes (Table 2). Using the Jaccard Similarity Tool in GW, we compared the conserved set of cisplatin-responsive genes with the differentially expressed genes. Table 2 shows the summary of these data. Our results indicated that of the 102 cisplatin-associated genes conserved in human, mouse and rat, 16 genes were up-regulated in at least one of the four subtypes and 11 were down-regulated in at least one subtype.

**Table 2:** This table shows the 20 genes that are in the set of conserved cisplatin-responsive gene set, and how those genes are up- and down-expressed in each of four Lehmann-identified TNBC subtypes. ‘UP’ indicates the gene is over-expressed and ‘DOWN’ indicates the gene is under-expressed. The ‘LAR’ or ‘M’ column indicates that the gene is differentially expressed in one of the two cisplatin-resistant subtypes compared with the BL1 or BL2 sensitive subtypes. The ‘Cell Death’ column indicates if the gene has been associated with a Gene Ontology term describing an aspect of cell death

Gene Symbol	Gene Name	BL1	BL2	M	LAR	Resistant	Death
*ABCC2*	ATP binding cassette subfamily C member 2				UP	*	
*ADM*	adrenomedullin		UP	UP			*
*AKT1*	AKT serine/threonine kinase 1				UP	*	*
*BCL2*	BCL2 apoptosis regulator	DOWN					*
*BCL2L1*	BCL2 like 1				UP	*	*
*CASP8*	caspase 8			DOWN	UP	*	*
*CAV1*	caveolin 1		UP				*
*CLU*	clusterin	DOWN			UP	*	*
*FAS*	Fas cell surface death receptor			DOWN		*	*
*FOS*	Fos proto-oncogene, AP-1 transcription factor subunit	DOWN					*
*GSR*	glutathione-disulfide reductase				UP	*	
*GJA1*	gap junction protein alpha 1	DOWN	UP	UP			*
*HSPB1*	heat shock protein family B (small) member 1		UP		UP		*
*MSH2*	mutS homolog 2	UP			DOWN	*	*
*NOX4*	NADPH oxidase 4	DOWN		UP		*	*
*NQO1*	NAD(P)H quinone dehydrogenase 1				UP	*	*
*PTK2*	protein tyrosine kinase 2	UP					*
*TUBA1A*	tubulin alpha 1a			UP	DOWN	*	
*VCAM1*	vascular cell adhesion molecule 1			DOWN		*	
*VIM*	vimentin			UP	DOWN	*	

Our results show that of the differentially expressed genes in each subtype, only a small proportion are associated with the set of cisplatin-interacting genes: BL1 (2:215 up and 5:251 down), BL2(4:154 up and 0:127 down), M(5:247 up and 3:446 down), and LAR (8:805 up and 3:382 down). If we examine only the set of genes that show different expression behavior in the resistant LAR and M subtypes when compared to the sensitive BL1 and BL2 subtypes, a signature of 13 genes is identified, shown in column 6 of Table 2. These results show that the differential expression of cisplatin-associated genes in breast cancer subtypes involves only a small percentage, 20 genes, of the overall genes used to characterize the subtypes and there is a set of 13 cisplatin-associated genes whose differential expression is characteristic of the two resistant subtypes.

The results of GO term enrichment analysis on the 20 differentially regulated genes for biological process are shown in Additional file 5: Table S5 [Additional file 5: Table S5]. Consistent with the conserved set of 102 cisplatin-associated genes, the 20 genes overlapping with the TNBC differentially regulated sets were also enriched for stress-response genes, aging, and genes that are involved in regulating programmed cell death. In addition terms representing the ‘CD95-death inducing complex’ and focal adhesion complexes were enriched, consistent with potential mechanisms of regulation of apoptosis and epithelial-to-mesenchymal transition mitochondrial outer membrane (*p* = 3.56e-05). Unlike the conserved set of genes, these 20 genes are not as significantly enriched for genes associated with telomeres (*p* = 1.1e-01) or nucleoplasm (*p* = 5.99e-02). This result shows that the subset of genes regulated in the TNBC subtypes are enriched for similar processes as the parental sets and are not biased towards other processes.

### Genes that are differentially regulated in cisplatin-resistant TNBC subtypes identify a variety of mechanisms to escape cisplatin toxicity

To try to understand whether the differential regulation of the 13 cisplatin-associated genes in the LAR and M subtypes could explain the subtype’s resistance, we examined each gene individually to determine if there was evidence that the over- or under-expression of these genes correlated with resistance to cisplatin. The results of our analysis are shown in Table 3, where the LAR and M subtypes are shown to vary in their signature of cisplatin genes that are differentially regulated. Seven of the genes are exclusively differentially expressed in the LAR subtype, three in the M subtype and three are differentially expressed in both subtypes. Interestingly, the direction of the differential expression for the three common genes is in opposite directions. Examining how these genes might influence cisplatin reistance shows that, while some of the genes influence apoptosis directly, others identify different upstream mechanisms of resistance. Since cisplatin is not a first-line treatment for TNBC, most studies correlating these genes with resistance or sensitivity to cisplatin are from other cancer types. Our results suggest that these genes may also influence cisplatin sensitivity in TNBC, and may help further elucidate the mechanisms of cisplatin action in TNBC and suggest more refined strategies for cisplatin treatment.

**Table 3:** This table shows genes that are differentially regulated when comparing the cisplatin-resistant versus cisplatin-sensitive TNBC subtypes. Column 2 is a brief note about the action of the gene. Column 3 is a representative reference supporting the mechanism

Gene Symbol	Evidence for Resistance	Reference
*ABCC2 (up in LAR)*	A transporter that when overexpressed results in cisplatin resistance	[42]
*AKT1 (up in LAR)*	A stress-response protein that when amplified or overexpressed is correlated with cisplatin resistance	[43, 44]
*BCL2L1 (up in LAR)*	Apoptosis-inhibitor, overexpression correlates with cisplatin resistance	[45, 46]
*CASP8 (up in LAR down in M)*	Required for cisplatin-associated apoptosis	[47, 48]
*CLU (up in LAR)*	Well known to contribute to chemoresistance including cisplatin	[49, 50]
*FAS* (down in M)	Overexpression induces cisplatin sensitivity and reduced expression correlates with resistance	[51–53]
*GSR (up in LAR)*	Involved in the detoxification of cisplatin	[54, 55]
*MSH2 (down in LAR)*	Required for cisplatin induced apoptosis	[56–58]
*NOX4 (up in M)*	Increased expression leads to more severe cisplatin toxicity	[37]
*NQO1 (up in LAR)*	A redox enzyme that has been show to contribute to resistance to cisplatin toxicity	[59–61]
*TUBA1A (up in M down in LAR)*	Correlated with cisplatin-reistance in esophageal cells	[62]
*VCAM1 (down in M)*	Associated with epithelial to mesenchymal transition overexpression contributes to cisplatin resistance	[63]
*VIM (up in M down in LAR)*	Associated with epithelial to mesenchymal transition	[64, 65]

## Discussion

We applied an integrated gene set analysis to identify potential biological mechanisms underlying cisplatin sensitivity in four different molecular subtypes of TNBC. We defined a set of 102 cisplatin-associated genes conserved across human, mouse, and rat, and we used knowledge about those genes to evaluate how those genes could be involved in the therapeutic response. Overall, our results show that many cisplatin-responsive genes are involved with the end stage of the effects of cisplatin treatment: cell death. Cell death is also the most globally differentially regulated process identified by cisplatin-responsive genes in all subtypes of TNBC. These results imply that agents that up-regulate apoptotic signaling, such as Trail sensitizers, should be investigated as effective global co-therapies for cisplatin treatment [66].

### Response to Cisplatin

To specifically investigate the differences in cisplatin response with respect to each of the subtypes, we examined the genes that were uniquely differentially expressed in the resistant LAR and M subtypes. Response to cisplatin can be modulated by a number of different mechanisms: decreased cellular import or increased cellular efflux of cisplatin, detoxification of cisplatin, defective DNA repair or resistance to cell cycle arrest or cell death [25, 67, 68].

As noted previously, Lehmann et al showed that in cell lines, the BL1 and BL2 subtypes often contained mutations in one of the *BRCA* genes. They hypothesized that the DNA repair defect explained why BL1 and BL2 are more sensitive to cisplatin than the M or LAR subtypes. It has recently been suggested that platins or PARP inhibitors are potential treatment options for TNBC with *BRCA* mutations [17]. A recent study by Zhao et al showed that other factors such as homologous recombination status may also influence cisplatin response in breast cancer [69]. Our work suggests that in addition to *BRCA* mutation status, other factors may contribute to differential sensitivity of these subtypes. As described above, our results show that cisplatin-associated genes involved in cell death are differentially expressed in all TNBC subtypes, but the LAR and M subtypes have a unique signature of genes that are not differentially regulated in the same way in the BL1 or BL2 subtypes.

**Fig. 4:**
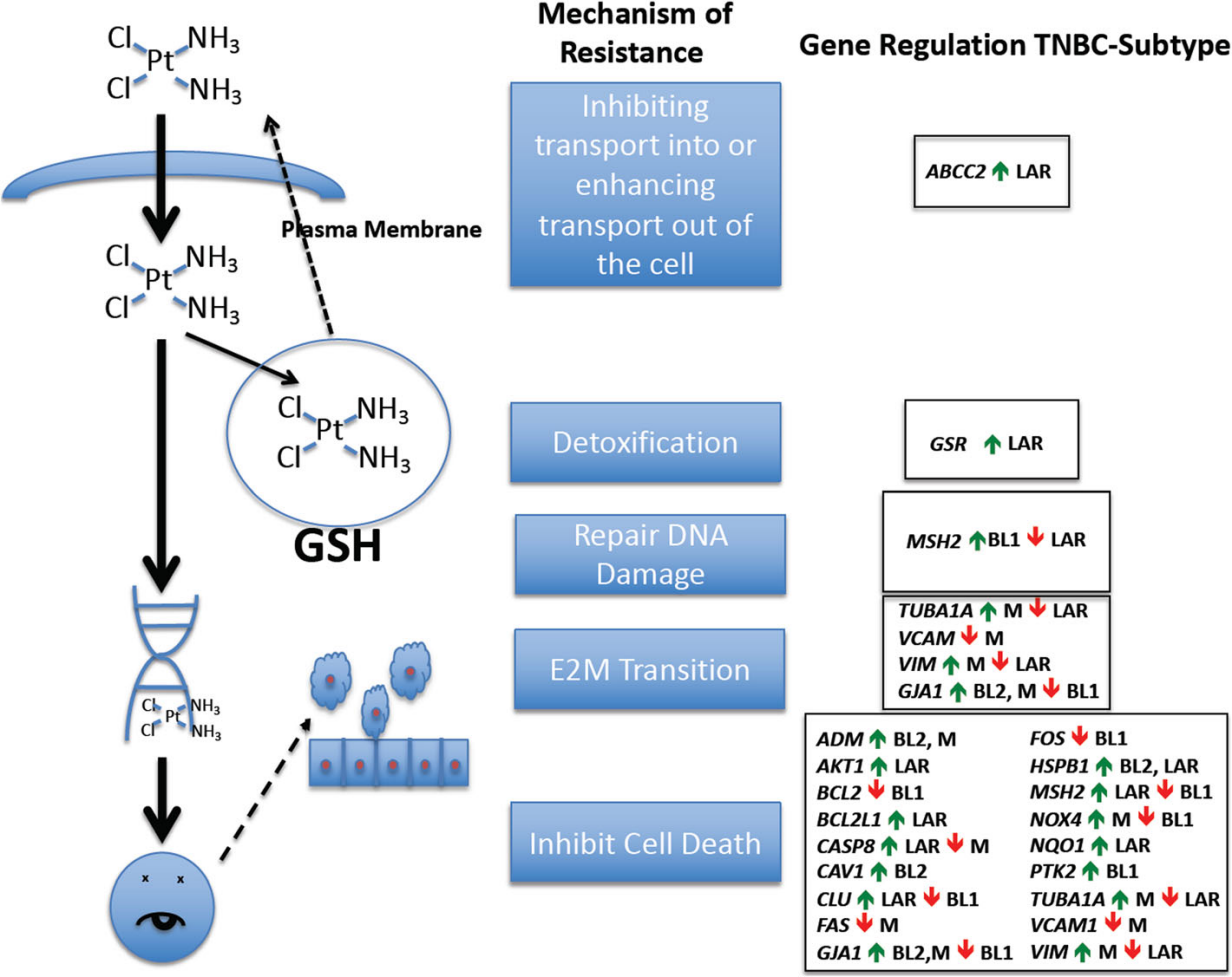
Title: Mechanisms of cisplatin-resistance in Four TNBC Subtypes. Legend: A schematic representation of the mechanisms by which a cell can become resistant to the effects of cisplatin, and genes that are involved in those processes. Regulation of the expression of genes and their direction of regulation is indicated for each of four TNBC subtypes described by Lehmann et al.

In particular, we find that the genes *ABCC2*, *AKT1*, *BCL2L1*, *CASP8, CLU*, *GSR*, *NQO1* are up-regulated in the LAR subtype and *MSH2* is downregulated. With the exception of *CASP8*, the regulation of all of these genes is consistent with reported resistance to cisplatin (Table 3). *ABCC2* and *GSR*, specifically, represent a transporter and a glutathione metabolic enzyme, respectively, that lie in a pathway that detoxifies and transports cisplatin out of the cell [42, 54]. The increase in *ABCC2* and *GSR*, and their actions upstream of the cell death related genes, provides us with a testable hypothesis for an additional mechanism that contributes to the relative cisplatin resistance of the LAR subtype compared to the other subtypes. That is to say, inhibition of either or both of these proteins could make LAR cells more sensitive to cisplatin treatment (Fig. 4). *AKT1*, *CLU* and *NQO1* encode proteins that respond to stress, including oxidative stress, which is one of the mechanisms of cisplatin action [70]. These three genes would contribute to cisplatin resitance in pathways downstream of *GSR* or *ABCC2* [43, 49, 59–61]. *BCL2L1* and *CASP8* are both proteins integral to the apoptotic program. *BCL2L1* is an inhibitor of apoptosis whose overexpression has been correlated with cisplatin-resistance, consistent with its upregulation in the resistant LAR subtype. The only down-regulated gene, *MSH2*, is a protein involved in DNA repair, although it has been shown to be necessary for the apoptotic action of cisplatin [56, 57]. The up-regulation of *CASP8* is counter-indicative of cisplatin resistance, since its overexpression has been shown to make cells more sensitive to cisplatin [47]. However, it is interesting to note that *CASP8* would lie the most downstream of all of the other genes that are differentially regulated in the LAR subtype and therefore may be epistatically masked by upstream changes.

The LAR subtype also shows differential regulation of some genes also differentially regulated in the M subtype, but neither of the basal subtypes. *VIM* and *TUBA1* are downregulated in the LAR subtype. Both *VIM* and *TUBA1* have previously been associated with cisplatin resistance, but the causal effect remains to be determined [62, 64, 65]. In ovarian cancer cells down-regulation of *VIM* expression resulted in resistance to cisplatin by potentially down-regulating its import and up-regulating its export, indicating that it might also be contributing to cisplatin resistance in the LAR subtype [64]. However, the factors controlling *VIM* expression and its exact role in cisplatin resistance in different cancer types are still not well understood. Some studies, including some breast-cancer studies show increased *VIM* expression correlates with cisplatin resistance [71–73]. One interesting question that arises from our analysis is whether or not the LAR subtype represents a heterogeneous population that can be further subdivided with respect to mechanisms of resistance and if so, what is the nature of the heterogeneity. Can some LAR tumors escape cisplatin by upregulating its transport out of the cell while others escape by different mechanisms such as upregulating *GSR*, or does a single tumor tend to accumulate multiple mechanisms of resistance? Because our analysis is retrospective and used aggregate data from previous studies, these types of questions require further investigation.

In the M subtype, some genes differentially regulated and potentially involved in cisplatin resistance differ from those identified in the LAR subtype. To fully understand the biology of cisplatin resistance in the M subtype, one area to further pursue is the epithelial-to-mesenchymal transition that results in increased *VIM* expression, which is downregulated in the LAR subtype.

The M subtype also shows differential up-regulation of *VIM*, *NOX4* and *TUBA1A*. *VCAM1* is downregulated in the M subtype. *VCAM1* has also been associated with an increase in epithelial-to-mesenchymal transition and has been correlated with resistance to cisplatin [63, 64]. Overexpression of *VCAM1* has been shown to confer cisplatin resistance in breast cancer cells [63]. The downregulation of *VCAM1* in the M subtype is counterintuitive to it being causative in this subtype’s lower sensitivity to cisplatin. As noted above, the expression of *VIM* is less well understood. Although overexpression correlates with cisplatin resistance in some contexts, it is still not well characterized mechanistically. At least two studies have shown that genes controlling the epithelial-to-mesenchymal transition, *ITGB1* and *TET1*, confer cisplatin resistance, and those genes also increase the expression of *VIM* [65, 73]. The gene sets of TNBC differentially expressed genes did not include *ITGB1* or *TET1*. *NOX4* is an NADPH oxidase that generates reactive oxygen species and can make the effects of cisplatin treatment more severe. However, overexpression of *NOX4* has been shown to result in normal breast cells being resistant to apoptosis [74]. Like *VCAM1*, the higher differential expression of *NOX4* is counterindictive for cisplatin resistance. *CASP8* is also downregulated in the M subtype. In contrast to LAR, downregulation of *CASP8* in the M subtype would lead to a defect in the apoptotic mechanism resulting in cisplatin resistance regardless of upstream triggers.

## Conclusions

We have used a gene-set comparative approach to study potential mechnisms of cisplatin resitance in TNBC subtypes. Out results show that in the resistant LAR subtype a small number of genes that are differentially expressed identify a variety of potential mechanisms that can be used to escape cisplatin toxicity; transport, detoxification, and direct and indirect involvement in programmed cell death. We hypothesize that the differential expression of these genes impacts how tumors of a given subtype will respond to the agent. In the resistant M subtype, a small number of genes is also differentially regulated, but the interpretation of their contribution to resistance is less clear. The differentially regulated genes in the M subtype identify the epithelial-to-mesenchymal transition and the control of reactive oxygen species as potential regulators of cisplatin response.

By focusing on genes known to be associated with cisplatin, our method identifies (or excludes) genes that can serve as a signature in the differential response of TNBC subtypes to cisplatin treatment. This gives an advantage over global gene expression classification systems in that we can pinpoint specific gene signatures that classify with respect to a targeted area of interest, in this case with cisplatin association. Our results suggest that additional therapies to enhance the apoptotic mechanism might be globally beneficial for the treatment of all types of TNBC, while the LAR subtype might benefit from a combination treatment of cisplatin and glutathione-modulator treatment agents [75]. For TNBC the analysis could be extended to investigate the molecular basis of the differences in response to other primary therapeutic agents such as taxane and anthracycline. One limitation to this extension is availability of data for analysis. These types of studies require existing experimental data with respect to response status and gene expression patterns for analysis and require high quality gene-chemical association data. In our study, we used existing data reported for TNBC subtypes and from the CTD resource to seed our analysis. As mentioned earlier, a limitation to this type of aggregate data is that it does not allow us to ask questions with respect to whether or not individual tumors or individual cells express different subsets of genes that confer resistance. These types of questions can be addressed in future studies in which wet-bench studies of expression from tumor samples or individual tumor cells are correlated with drug resitance or sensitivity and are analyzed in the context of high quality curated data about gene-chemical interactions. Ideally, a prospective strategy using markers such as *BRCA* status or PD-L1 to predict response-type would be most useful in deciding treatment options [17]. Our results identify genes that can be further studied as useful biomarkers.

## Supplementary information

**Additional file 1: Table S1.** Gene Ontology Terms enriched in the 102 cisplatin-associated genes Description of data: The VLAD graphical output for GO Biological Process and GO Cellular Component was examined and reported in tabular format. The five most specific terms and their respective p-values are listed. The analysis was run on September 2, 2019. The UniProt-GOA gene annotation data used was dated from February 26, 2018.

**Additional file 2: Table S2.** KEGG-Disease analysis of 102 cisplatin associated genes Description of data: A list of the disease categories in KEGG that were associated with the 102 cisplatin-associated genes. Gene symbols were used with default parameters in the Organism-specific search mode (hsa). The analysis was performed on Sept 6, 2019. Categories with at least 2 genes are shown.

**Additional file 3: Table S3.** KEGG-Pathways analysis of 102 cisplatin associated genes Description of data: A list of the pathway categories in KEGG that were associated with the 102 cisplatin-associated genes. Gene symbols were used with default parameters in the Organism-specific search mode (hsa). The analysis was performed on Sept 6, 2019. Categories with > 10 genes are shown.

**Additional file 4: Table S4.** String network analysis of 102 cisplatin associated genes Description of data: A list of GO terms and KEGG categories identified using the String network analysis tool with respective False-discovery rates. The analysis was performed on Sept 8, 2019.

**Additional file 5: Table S5.** Gene Ontology Terms enriched in the 20 cisplatin-associated genes Description of data: The VLAD graphical output for GO Biological Process and GO Cellular Component was examined and reported in tabular format. The five most specific terms and their respective *p*-values are listed. The analysis was run on September 2, 2019. The UniProt-GOA gene annotation data used was dated from February 26, 2018.

## Data Availability

The datasets generated and/or analysed during the current study are available in the GW repository [33].

## Abbreviations

BL1: Basal-like 1 subtype of Triple Negative Breast Cancer
BL2: Basal-like 2 subtype of Triple Negative Breast Cancer
ChEBI: Chemicals of Biological Interest
GO: Gene Ontology
GW: GeneWeaver
IM: Immunomodulatory subtype of Triple Negative Breast Cancer
LAR: Luminal androgen receptor subtype of Triple Negative Breast Cancer
M: Mesenchymal subtype of Triple negative Breast Cancer
MSL: Mesenchymal stem-like subtype of Triple Negative Breast Cancer
TNBC: Triple Negative Breast Cancer
VLAD: Visual Annotation Display
