# Supplementary material for: Cisplatin-resistant triple-negative breast cancer subtypes: multiple mechanisms of resistance
Source: BMC Cancer. 2019 Nov 4;19:1039. doi: 10.1186/s12885-019-6278-9 (PMC6829976; doi:10.1186/s12885-019-6278-9)
Supplement: Supplementary file 1 — Additional file 1: Table S1. Gene Ontology Terms enriched in the 102 cisplatin-associated genes Description of data: The VLAD graphical output for GO Biological Process and GO Cellular Component was examined and reported in tabular format. The five most specific terms and their respective p-values are listed. The analysis was run on September 2, 2019. The UniProt-GOA gene annotation data used was dated from February 26, 2018. [file 12885_2019_6278_MOESM1_ESM.docx]

| **GO Biological Process** | **GO Cellular Component** |
| --- | --- |
| response to oxygen-containing compound (p=5.67e-38) | nucleoplasm (p=5.5e-12) |
| regulation of apoptotic process (1.29-35) | cytosol (p=2.5e-11) |
| cellular response to oxidative stress (p=1.43e-34) | chromosome, telomeric region (p=3.3e-10) |
| aging (p=1.32e-34) | mitochondrion (p=2.6e-08) |
| apoptotic Process (p=1.8e-32) | extracellular space (p=3.7e-07) |
| response to ionizing radiation (p=7.49e-32) | membrane raft (p=7.1e-07) |
