# Supplementary material for: Cisplatin-resistant triple-negative breast cancer subtypes: multiple mechanisms of resistance
Source: BMC Cancer. 2019 Nov 4;19:1039. doi: 10.1186/s12885-019-6278-9 (PMC6829976; doi:10.1186/s12885-019-6278-9)
Supplement: Supplementary file 2 — Additional file 2: Table S2. KEGG-Disease analysis of 102 cisplatin associated genes Description of data: A list of the disease categories in KEGG that were associated with the 102 cisplatin-associated genes. Gene symbols were used with default parameters in the Organism-specific search mode (hsa). The analysis was performed on Sept 6, 2019. Categories with at least 2 genes are shown. [file 12885_2019_6278_MOESM2_ESM.docx]

| **KEGG Identifier** | **Disease Name** | **# of Genes** |
| --- | --- | --- |
| H00017 | Esophageal cancer | 5 |
| H00084 | Graft-versus-host disease | 4 |
| H00025 | Penile cancer | 4 |
| H00005 | Chronic lymphocytic leukemia | 4 |
| H00403 | Disorders of nucleotide excision repair | 3 |
| H00108 | Autoimmune lymphoproliferative syndromes | 3 |
| H00083 | Allograft rejection | 3 |
| H00030 | Cervical cancer | 3 |
| H00028 | Choriocarcinoma | 3 |
| H00020 | Colorectal cancer | 3 |
| H01227 | Inflammatory bowel disease IBD | 2 |
| H01709 | Glucocorticoid-induced osteonecrosis | 2 |
| H01667 | Medulloblastoma | 2 |
| H01555 | Merkel cell carcinoma | 2 |
| H01529 | Avascular necrosis of femoral head | 2 |
| H01470 | Giant cell tumor of bone | 2 |
| H01464 | Mantle cell lymphoma | 2 |
| H01463 | Mycosis fungoides | 2 |
| H00881 | Li-Fraumeni syndrome | 2 |
| H00604 | Deafness, autosomal dominant | 2 |
| H00055 | Laryngeal cancer | 2 |
| H00048 | Hepatocellular carcinoma | 2 |
| H00046 | Cholangiocarcinoma | 2 |
| H00042 | Glioma | 2 |
| H00041 | Kaposi sarcoma | 2 |
| H00040 | Squamous cell carcinoma | 2 |
| H00037 | Rhabdomyosarcoma | 2 |
| H00036 | Osteosarcoma | 2 |
| H00032 | Thyroid cancer | 2 |
| H00031 | Breast cancer | 2 |
| H00027 | Ovarian cancer | 2 |
| H00022 | Bladder cancer | 2 |
| H00016 | Oral cancer | 2 |
| H00013 | Small cell lung cancer | 2 |
| H00010 | Multiple myeloma | 2 |
| H00009 | Adult T-cell leukemia | 2 |
| H00006 | Hairy cell leukemia | 2 |
