# Supplementary material for: Cisplatin-resistant triple-negative breast cancer subtypes: multiple mechanisms of resistance
Source: BMC Cancer. 2019 Nov 4;19:1039. doi: 10.1186/s12885-019-6278-9 (PMC6829976; doi:10.1186/s12885-019-6278-9)
Supplement: Supplementary file 3 — Additional file 3: Table S3. KEGG-Pathways analysis of 102 cisplatin associated genes Description of data: A list of the pathway categories in KEGG that were associated with the 102 cisplatin-associated genes. Gene symbols were used with default parameters in the Organism-specific search mode (hsa). The analysis was performed on Sept 6, 2019. Categories with > 10 genes are shown. [file 12885_2019_6278_MOESM3_ESM.docx]

| **ID** | **Name** | **# of Genes** |
| --- | --- | --- |
| hsa05200 | Pathways in cancer - Homo sapiens | 36 |
| hsa04210 | Apoptosis - Homo sapiens | 27 |
| hsa05161 | Hepatitis B - Homo sapiens | 23 |
| hsa05170 | Human immunodeficiency virus 1 infection - Homo sapiens | 22 |
| hsa01524 | Platinum drug resistance - Homo sapiens | 22 |
| hsa05169 | Epstein-Barr virus infection - Homo sapiens | 21 |
| hsa05166 | Human T-cell leukemia virus 1 infection - Homo sapiens | 21 |
| hsa05165 | Human papillomavirus infection - Homo sapiens | 21 |
| hsa04218 | Cellular senescence - Homo sapiens | 21 |
| hsa05210 | Colorectal cancer - Homo sapiens | 20 |
| hsa05163 | Human cytomegalovirus infection - Homo sapiens | 20 |
| hsa04115 | p53 signaling pathway - Homo sapiens | 20 |
| hsa05206 | MicroRNAs in cancer - Homo sapiens | 19 |
| hsa05167 | Kaposi sarcoma-associated herpesvirus infection - Homo sapiens | 19 |
| hsa04933 | AGE-RAGE signaling pathway in diabetic complications - Homo sapiens | 19 |
| hsa05162 | Measles - Homo sapiens | 18 |
| hsa05152 | Tuberculosis - Homo sapiens | 17 |
| hsa05130 | Pathogenic Escherichia coli infection - Homo sapiens | 17 |
| hsa04151 | PI3K-Akt signaling pathway - Homo sapiens | 17 |
| hsa04068 | FoxO signaling pathway - Homo sapiens | 17 |
| hsa04010 | MAPK signaling pathway - Homo sapiens | 17 |
| hsa05418 | Fluid shear stress and atherosclerosis - Homo sapiens | 16 |
| hsa05142 | Chagas disease | 16 |
| hsa04932 | Non-alcoholic fatty liver disease | 16 |
| hsa04668 | TNF signaling pathway - Homo sapiens | 16 |
| hsa05225 | Hepatocellular carcinoma - Homo sapiens | 15 |
| hsa05222 | Small cell lung cancer - Homo sapiens | 15 |
| hsa05205 | Proteoglycans in cancer - Homo sapiens | 15 |
| hsa05160 | Hepatitis C - Homo sapiens | 15 |
| hsa05145 | Toxoplasmosis - Homo sapiens | 15 |
| hsa04625 | C-type lectin receptor signaling pathway - Homo sapiens | 15 |
| hsa05212 | Pancreatic cancer - Homo sapiens | 14 |
| hsa05203 | Viral carcinogenesis - Homo sapiens | 14 |
| hsa05168 | Herpes simplex virus 1 infection - Homo sapiens | 14 |
| hsa04110 | Cell cycle - Homo sapiens | 14 |
| hsa01522 | Endocrine resistance - Homo sapiens | 14 |
| hsa05220 | Chronic myeloid leukemia - Homo sapiens | 13 |
| hsa05164 | Influenza A - Homo sapiens | 13 |
| hsa05133 | Pertussis - Homo sapiens | 13 |
| hsa04657 | IL-17 signaling pathway - Homo sapiens | 13 |
| hsa04621 | NOD-like receptor signaling pathway - Homo sapiens | 13 |
| hsa05226 | Gastric cancer - Homo sapiens | 12 |
| hsa05215 | Prostate cancer - Homo sapiens | 12 |
| hsa05135 | Yersinia infection - Homo sapiens | 12 |
| hsa04620 | Toll-like receptor signaling pathway - Homo sapiens | 12 |
| hsa04510 | Focal adhesion - Homo sapiens | 12 |
| hsa05224 | Breast cancer - Homo sapiens | 11 |
| hsa05202 | Transcriptional misregulation in cancer - Homo sapiens | 11 |
| hsa05140 | Leishmaniasis - Homo sapiens | 11 |
| hsa05014 | Amyotrophic lateral sclerosis | 11 |
| hsa04722 | Neurotrophin signaling pathway - Homo sapiens | 11 |
| hsa04215 | Apoptosis - multiple species - Homo sapiens | 11 |
| hsa04071 | Sphingolipid signaling pathway - Homo sapiens | 11 |
| hsa04066 | HIF-1 signaling pathway - Homo sapiens | 11 |
| hsa01100 | Metabolic pathways - Homo sapiens | 11 |
