# Supplementary material for: Cisplatin-resistant triple-negative breast cancer subtypes: multiple mechanisms of resistance
Source: BMC Cancer. 2019 Nov 4;19:1039. doi: 10.1186/s12885-019-6278-9 (PMC6829976; doi:10.1186/s12885-019-6278-9)
Supplement: Supplementary file 4 — Additional file 4: Table S4. String network analysis of 102 cisplatin associated genes Description of data: A list of GO terms and KEGG categories identified using the String network analysis tool with respective False-discovery rates. The analysis was performed on Sept 8, 2019. [file 12885_2019_6278_MOESM4_ESM.docx]

| **GO Term** | **False Discovery Rate** |
| --- | --- |
| response to stress | 3.00E-47 |
| response to abiotic stimulus | 2.45E-45 |
| cellular response to stress | 1.12E-43 |
| response to oxygen-containing compound | 1.67E-39 |
| response to chemical | 3.72E-38 |
| cellular response to chemical stimulus | 1.45E-37 |
| regulation of apoptotic process | 1.17E-33 |
| response to oxidative stress | 1.17E-33 |
| response to drug | 1.49E-33 |
| regulation of cell death | 5.09E-33 |
| cellular response to oxidative stress | 6.08E-32 |
| positive regulation of metabolic process | 6.08E-32 |
| response to toxic substance | 6.08E-32 |
| response to radiation | 6.08E-32 |
| response to organic substance | 6.47E-32 |
| aging | 6.97E-32 |
| cellular response to stimulus | 7.53E-32 |
| positive regulation of nitrogen compound metabolic process | 7.80E-32 |
| positive regulation of macromolecule metabolic process | 1.07E-31 |
| response to external stimulus | 1.60E-31 |
| **KEGG Pathway** | **False Discovery Rate** |
| Apoptosis | 7.68E-30 |
| Platinum drug resistance | 1.54E-27 |
| Pathways in cancer | 5.48E-26 |
| Hepatitis B | 3.39E-25 |
| Colorectal cancer | 4.57E-23 |
| p53 signaling pathway | 4.88E-23 |
| AGE-RAGE signaling pathway in diabetic complications | 3.57E-22 |
| Cellular senescence | 1.14E-21 |
| HTLV-I infection | 8.37E-18 |
| MicroRNAs in cancer | 8.54E-18 |
| Kaposi's sarcoma-associated herpesvirus infection | 1.02E-17 |
| Chagas disease (American trypanosomiasis) | 1.51E-17 |
| FoxO signaling pathway | 2.01E-17 |
| TNF signaling pathway | 3.41E-17 |
| Small cell lung cancer | 1.07E-16 |
| Human papillomavirus infection | 4.39E-16 |
| Fluid shear stress and atherosclerosis | 5.70E-16 |
| Toxoplasmosis | 8.82E-16 |
| Tuberculosis | 1.04E-15 |
| Non-alcoholic fatty liver disease (NAFLD) | 2.52E-15 |
