# Supplementary material for: Cisplatin-resistant triple-negative breast cancer subtypes: multiple mechanisms of resistance
Source: BMC Cancer. 2019 Nov 4;19:1039. doi: 10.1186/s12885-019-6278-9 (PMC6829976; doi:10.1186/s12885-019-6278-9)
Supplement: Supplementary file 5 — Additional file 5: Table S5. Gene Ontology Terms enriched in the 20 cisplatin-associated genes Description of data: The VLAD graphical output for GO Biological Process and GO Cellular Component was examined and reported in tabular format. The five most specific terms and their respective p-values are listed. The analysis was run on September 2, 2019. The UniProt-GOA gene annotation data used was dated from February 26, 2018. [file 12885_2019_6278_MOESM5_ESM.docx]

| **GO Biological Process** | **GO Cellular Component** |
| --- | --- |
| response to stress (p=5.5e-13) | Plasma membrane bounded cell projection (p=1.28e-06) |
| response to oxygen-containing compound (2.18e-11) | focal adhesion (p=2.49e-6) |
| regulation of cell death (p=8.66e-11) | CD95 death-inducing signaling complex (p=1.62e-05) |
| aging (p=1.8e-10) | mitochondrial outer membrane (p=3.56e-05) |
| response to metal ion (p=1.64e-09) | cytosol (p=7.21e-05) |
